# Exploring the Impact of Affinity and Unpleasantness on Conditioned Pain Modulation among Healthy Individuals

**DOI:** 10.3390/biomedicines12061172

**Published:** 2024-05-25

**Authors:** María del Rocío Ibancos-Losada, Ángeles Díaz-Fernández, Irene Cortés-Pérez, Esteban Obrero-Gaitán, Virginia López-Moreno, María Catalina Osuna-Pérez

**Affiliations:** 1 Department of Health Sciences, University of Jaen, Campus las Lagunillas, 23071 Jaen, Spain; mibancos@ujaen.es (M.d.R.I.-L.); icortes@ujaen.es (I.C.-P.); eobrero@ujaen.es (E.O.-G.); vlmfisioterapia@gmail.com (V.L.-M.); mcosuna@ujaen.es (M.C.O.-P.); 2 FisioMas Physiotherapy Center, C/Antonio Sánchez Bonil 4, Peal de Becerro, 23460 Jaen, Spain

**Keywords:** conditioning stimulus, affinity, unpleasantness, conditioned pain modulation, pain modulation

## Abstract

The variability of the Conditioned Pain Modulation (CPM) effect can be attributed to conditioning stimulus (CS) characteristics, such as intensity, duration, unpleasantness, or affinity. This study investigates the impact of affinity and unpleasantness variables on the CPM effect using two protocols (cold water and ischemia) in the same healthy individuals (n = 54). Additional variables were also examined for their potential influence on the CPM effect. The main results are as follows: (1) a higher level of affinity and a lower level of unpleasantness for the stimuli used resulted in a stronger CPM effect; (2) significant differences were observed in the extreme categories (high and low) of both variables, whereas the ‘indifferent’ group did not show a clear trend; (3) within-subject analysis demonstrated that affinity for the CS had a clear impact on the CPM effect; (4) no correlations were found between the CPM effect and the additional variables, except for the extraversion variable with the CPM effect of the ischemia protocol, and CS duration variable with CPM effect in the cold water protocol; and (5) only the affinity variable explained the CPM effect in both protocols in the multiple linear regression analysis. The affinity variable was found to influence the CPM effects significantly, indicating its important role in our perception and response to pain.

## 1. Introduction 

Conditioned Pain Modulation (CPM) is an endogenous pain-inhibitory pathway in humans that aims to reduce or inhibit pain [1]. It is closely associated with a phenomenon called Diffuse Noxious Inhibitory Control (DNIC), which was initially studied in rats by Le Bars [2]. The mechanism of action can be summarized as ‘pain inhibits pain’ [3], meaning that a painful stimulus applied to one part of the body can inhibit pain in another part of the body by activating this phenomenon [4]. CPM has gained importance in recent years due to its clinical applications. Specifically, experimental protocols can be used to evaluate the performance of this inhibition pathway [5]. Multiple studies have suggested that individuals with chronic pain may have altered CPM pathways, resulting in a lower CPM effect compared to healthy individuals [6].

Research has highlighted the variability of the CPM effect both within and between individuals. This variability can be attributed to personal characteristics such as sex or age [7], socioeconomic factors like economic and educational levels [7,8], lifestyle choices including alcohol consumption or sleep patterns [9], and cognitive–emotional states such as anxiety, depression, or catastrophizing. Notably, higher scores in these psychological variables correlate with a deficient CPM effect [10]. Conversely, positive attributes like resilience, optimism, and positive expectations have increased the CPM effect [11,12,13].

Moreover, the conditioning stimulus (CS) characteristics, such as intensity, duration, unpleasantness, salience, or affinity, also impact the CPM effect. While the relationship between CS intensity and the CPM effect is well-documented [14,15,16], the influence of salience or unpleasantness is less understood. A recent study investigated the attribute of pain unpleasantness and the impact on the CPM effect, finding that lower CS unpleasantness was associated with an improved CPM effect [11]. Only one previous study has explored the impact of affinity towards the CS, revealing that individuals with a preference for cold water as a CS exhibited a more pronounced CPM effect than those without such affinity [17].

Given these findings, research into the variables influencing the CPM effect is necessary. Understanding these factors can enhance our knowledge of pain modulation processes and suggest that treatments aligning with patient preferences and managing psychological factors could improve the CPM effect and clinical outcomes in chronic pain sufferers [10]. This study hypothesized that the CPM effect is modulated by affinity and unpleasantness due to CS applied. It postulates that individuals with higher affinity or lower unpleasantness towards the CS will exhibit a greater CPM effect. The research investigates the impact of affinity and unpleasantness on the CPM effect, using cold water and ischemic pressure conditioning stimulations with a cross-over design among healthy participants.

## 2. Materials and Methods

### 2.1. Participants

Non-probabilistic sampling was used to recruit participants through advertisements on various social networks. Those who met the criteria were eligible to participate. The study was conducted following the Declaration of Helsinki, good clinical practices, and all applicable laws and regulations. The Ethics Committee of the University of Jaén approved the research (ABR.23/2 INV). All the participants received the information about the study and provided written informed consent to participate. The inclusion criteria were (1) healthy volunteers, (2) being over 18 years old, and (3) having knowledge and understanding of the Spanish language. Participants who were pregnant, taking any pain medication, drugs, or alcohol 24 h before the study, or experiencing any pain or disease at the time or one week before were excluded from the study.

### 2.2. Procedures

All participants completed the Spanish versions of several questionnaires (details of the questionnaires are below), and they provided information on their sex (male/female), age (in years), and BMI (in kg/m^2^) using a self-developed questionnaire.

The participants also completed two CPM protocols in which the test stimulus (TS) of algometry was combined with the conditioning stimuli (CS) of cold water or ischemic pressure. The protocols, the ‘Cold Water Protocol’ and the ‘Ischemia Protocol’, were conducted on two different days with an exactly one-week gap between them. The order in which the protocols were administered was randomized using the Epidat 3.1 program (Department of Health, Santiago de Compostela, Galicia, Spain). Therefore, the participants could belong to Group A + B (first day—cold water protocol, second day—ischemia protocol) or Group B + A (first day—ischemia protocol, second day—cold water protocol). The study took place at the University of Jaén from May to June 2023. Each intervention lasted approximately 30–45 min. The two protocols followed the same methodological sequence.

#### 2.2.1. Application of the ‘Familiarization Test’

The familiarization test is the initial test aimed at familiarizing the participant with the pressure of the algometer, allowing the participant to better discriminate and evaluate sensations during the study. The investigator applied pressure progressively at three different points [15,18] in the neck region and always in the same order (two points at the superior fibers of the trapezius and one point on the scalene muscle) with a digital algometer (FPIX; Wagner Instruments, Greenwich, CT, USA) on the non-dominant side. For each point, the participant indicated when the pressure was perceived as painful (pressure pain threshold, PPT). The investigator stopped applying pressure and recorded the pressure reading on the algometer (kg/cm^2^) [19].

#### 2.2.2. Application of the TS (20 min Later)

The investigator performed the same procedure but on the dominant side. The mean of the three PPTs was calculated, and this mean value was considered the Pre-conditioning stimulus variable (Pre-CS).

#### 2.2.3. Application of the CS

- Cold Water Protocol: The participants remained seated and, on receiving the signal, were requested to submerge their non-dominant foot (three centimeters above the lateral malleolus) [17,20] in a container of cold water (8–10 °C) [17,19]. A water thermometer controlled the temperature, and the water was maintained within this range by the container’s cooling system. Every 30 s, the participants were asked to assess the pain sensation produced by the cold water. If it scored 6/10 NRS, they would remove their foot from the water; if not, the foot was submerged until 6/10 NRS was reached [17,21]. The participants could also indicate that they had reached 6/10 NRS before being asked every 30 s. Once this score was reached, the foot was removed from the water, and the TS was applied consecutively (sequential paradigm). The time was timed (seconds), and the maximum time in the water (for safety) was two minutes.
- Ischemia Protocol: Ischemia was caused by a blood pressure cuff placed at the ankle of the non-dominant leg and was inflated to a pressure of 250–260 mmHg [17,21]. The participants were seated with their leg raised for one minute [17,21]. Following this minute, the participants were asked to assess the pain sensation produced by the ischemia every 30 s. If the pain sensation reached 6/10 on the NRS, this part of the test was ended; if not, they had to flex and extend their ankle until this score on the NRS was reached [17,21]. Once 6/10 was reached on the NRS, the cuff was deflated, and the participants lowered their leg (sequential paradigm). Again, the participants could indicate they had reached 6/10 NRS before asking every 30 s. This time was timed (seconds).

#### 2.2.4. Application of the Second TS (Sequential Paradigm)

Again, the participants were asked to indicate when the pressure was PPT for each of the three points on the neck. The mean of the three values was recorded and considered the Post-conditioning stimulus variable (Post-CS).

### 2.3. Variables

#### 2.3.1. CPM Effect

CPM was assessed by calculating the PPT difference between the second TS and the first TS (Post-CS minus Pre-CS) in each protocol. This yielded two variables: ‘CPM effect Ischemia Protocol’ and ‘CPM effect Cold Water Protocol’. A positive CPM effect indicated decreased pain, indicating that the CPM phenomenon had been triggered. Conversely, a negative result indicated increased pain, suggesting that the CPM phenomenon had not been activated [20,22].

#### 2.3.2. Affinity to Stimulus Applied

The participants’ affinity to the stimulus was determined by their personal preference, which was based on their previous life experiences with that stimulus. Before starting the CPM protocols, the participants rated their affinity for each stimulus (cold water or ischemia) on a scale of 0 to 10, with 0 indicating no affinity and 10 as the highest. For an accurate assessment, examples were provided. For cold water, its perception in daily scenarios like river bathing, and for ischemia, the sensation from a sphygmomanometer cuff. Affinity was classified into three categories [23]: ‘low’ (0–3), indicating minimal liking; ‘indifferent’ (4–6), showing ambivalence; and ‘high’ (7–10), denoting strong liking. This question was included in the questionnaire, and the participant had to write down the score on it to prevent the researcher who carried out the CPM protocol from being influenced by the participant’s response.

#### 2.3.3. Unpleasantness Due to Stimulus Applied

After completing the experimental protocols, participants were asked to rate their unpleasantness on a scale of 0 to 10 for each CS (cold water and ischemia), where 0 meant not unpleasant and 10 extremely unpleasant. Unpleasantness ratings fell into three categories [23]: ‘low’ (0–3), indicating the stimulus was slightly unpleasant; ‘indifferent’ (4–6), denoting the stimulus was painful but neither slightly nor highly unpleasant; and ‘high’ (7–10), suggesting the stimulus was perceived as highly unpleasant.

#### 2.3.4. Anxiety and Depression

These variables were evaluated using the Spanish version of the Hospital Anxiety and Depression Scale (HADS) [24]. The HADS is a 14-item scale, with 7 items each for the anxiety and depression subscales. The scale uses a Likert-type response format, with scores ranging from 0 to 3 points. The scale provides two total scores, one for anxiety and one for depression, ranging from 0 to 21 on each subscale. Higher scores indicate higher levels of anxiety and depressive symptoms. The Cronbach’s alpha coefficients for the anxiety and depression subscales are 0.85 and 0.84, respectively.

#### 2.3.5. Stress

Stress was measured using the Spanish version of the Perceived Stress Scale (PSS) [25]. The self-administered scale consists of 14 items, a 5-point Likert response ranging from 0 (never) to 4 (very often). Items 4, 5, 6, 7, 9, 10, and 13 are in reverse order, which is significant to consider for their interpretation. Scores from 0 to 56 can be obtained, where a higher score corresponds to a higher level of perceived stress. It shows adequate internal consistency (α = 0.81) and concurrent validity and sensitivity.

#### 2.3.6. Catastrophizing

Catastrophizing was measured using the Spanish version of the Pain Catastrophizing Scale (PCS) [26]. This is a 13-item self-administered questionnaire in which subjects indicate the degree to which they experience each of the thoughts or feelings using a 5-point Likert-type scale ranging from 0 (never) to 4 (always). A total score is obtained from the scale that reflects the level of catastrophizing in the subject’s pain. Low scores indicate a low level of catastrophizing, and high values indicate a high level of catastrophizing in the subject. This scale comprises three dimensions: (a) rumination, (b) magnification, and (c) helplessness. The Cronbach’s alpha coefficient is 0.79.

#### 2.3.7. Kinesiophobia

Kinesiophobia was measured using the Spanish version of the Tampa Kinesiophobia Scale (TSK) [27]. Kinesiophobia is defined as the fear of moving due to pain or fear of injury. This questionnaire is made up of 11 items. The response options are 4-point Likert type ranging from 1 (totally disagree) to 4 (totally agree). The higher the score, the higher the level of kinesiophobia. Adequate internal consistency (α = 0.79).

#### 2.3.8. Resilience

Resilience was measured using the Spanish version of the Brief Resilience Scale (BRS) [28]. Resilience is defined as the ability to bounce back from stressful circumstances. The questionnaire consists of 6 items where this capacity is measured. Each item is scored on a 4-point Likert-type scale ranging from 1 (strongly disagree) to 4 (strongly agree). It is important for the interpretation to know that items 1, 3, and 5 are in the direct sense while items 2, 4, and 6 are in the reverse sense. The higher the score, the greater the resilience. Adequate internal consistency (α = 0.83), convergent, concurrent and predictive validity.

#### 2.3.9. Personality

Personality was measured using the Spanish version of the Revised and Abbreviated Eysenck Personality Questionnaire (EPQR-A) [29]. This questionnaire is made up of 24 items that allow evaluating four subscales (6 items each): extraversion (items 2, 4, 13, 15, 20, and 23), neuroticism (items 1, 9, 11, 14, 18, and 21), psychoticism (items 3, 6, 8, 12, 16, and 22) and sincerity (items 5, 7, 10, 17, 19, and 24). The response format is yes (1) or no (0), scoring each subscale between 0 and 6 and being more similar to that trait or personality. Adequate internal consistency (α > 0.54) and validity.

### 2.4. Statistical Analysis

Data were processed using SPSS version 21.00 (SPSS Inc., Chicago, IL, USA). A significance threshold was set at *p* < 0.05, with a 95% confidence interval [30]. The Kolmogorov–Smirnov test assessed the normality of the distribution of data. Descriptive statistics were computed, including means and standard deviations for continuous variables and frequencies and percentages for categorical variables.

To evaluate the influence of affinity and unpleasantness variables on the CPM effect across both protocols, one-way ANOVA and post-hoc Bonferroni tests were employed.

Paired-sample *t*-tests were also conducted to examine intra-subject CPM effects, categorizing subjects based on their affinity for the applied stimuli. This categorization resulted in four groups: (1) subjects with a high affinity for cold water stimulus and low or indifferent affinity for ischemic stimulus, (2) subjects with a low or indifferent affinity for cold water stimulus and high affinity for ischemic stimulus, (3) subjects with high affinity for both stimuli, and (4) subjects with low or indifferent affinity for both stimuli. A similar grouping and analysis were applied to the variable unpleasantness.

The Pearson correlation coefficient was calculated to determine the linear relationship between the CPM effect for both the ischemia and cold water protocols and additional variables such as anxiety, depression, stress, catastrophizing, kinesiophobia, resilience, personality traits, and duration of the CS (in both protocols). Additionally, correlations between CS duration and variables such as affinity and unpleasantness were also explored. This correlation coefficient was considered ‘strong’ if it was >0.50, ‘moderate’ between 0.30 and 0.50, and ‘weak’ if it was <0.30.

A linear regression analysis was performed with the studied variables to establish the percentage of variance explained in the dependent variable, the CPM effect, for both the ischemia and cold water protocols.

## 3. Results

**Figure 1:**
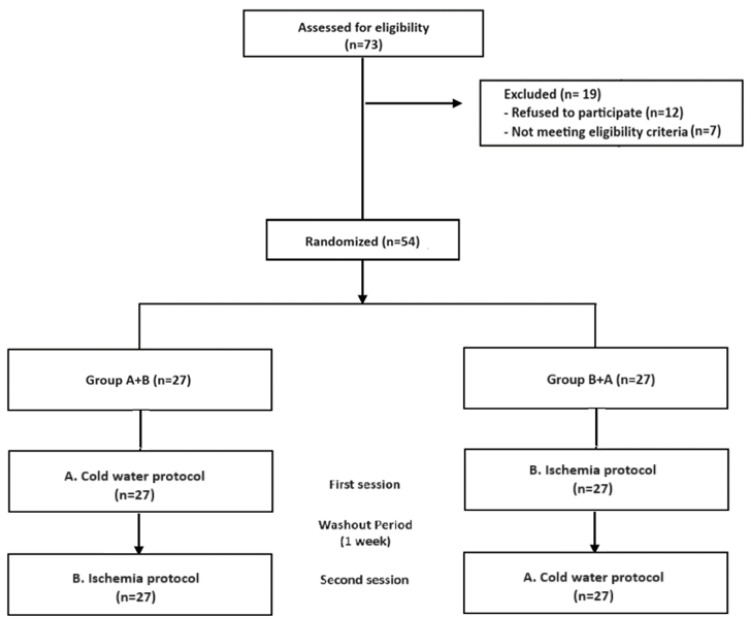
Flow-chart.

A total of 73 people responded to the advertisements. Twelve of them declined to participate because they were not interested in pain-related studies or had scheduling conflicts. Seven other participants did not meet the requirements for participation. Therefore, 54 healthy participants were enrolled in the study, and none of them dropped out during the study (Figure 1).

The sample primarily consisted of women (68.8%) with an average age of 39.15 ± 13.81 years and a BMI of 24.19 ± 3.89. Participants exhibited low levels of depression, anxiety, catastrophizing, stress, and kinesiophobia and had a high level of resilience. They also showed low levels of psychoticism, neuroticism, and sincerity and high levels of extraversion. The ‘Cold Water Protocol’ and the ‘Ischemia Protocol’ had inhibitory effects, with CPM effects of 0.16 ± 0.31 and 0.17 ± 0.30, respectively. The average duration of the cold water CS was 68.65 ± 39.34 s, while the ischemia CS lasted 121.26 ± 41.30 s. Further details can be found in Table 1, where descriptive characteristics of the sample and protocols are provided.

The study found a relationship between the CPM effect and the affinity variable. Specifically, a higher affinity for the applied stimulus is associated with a stronger CPM effect. On the other hand, there is an inverse relationship with the unpleasantness variable, where a higher unpleasantness for the applied stimulus is associated with a weaker CPM effect. The Bonferroni post-hoc test revealed significant differences between the extreme ‘high’ and ‘low’ categories for both the affinity and unpleasantness variables. However, the ‘indifferent’ group did not show a clear trend. The results of these analyses can be found in Table 2.

When studying the affinity variable at an intra-subject level, the paired samples *t*-test found that 44.44% of the participants had opposite affinities to the applied CS (low/indifferent affinity to one CS and high affinity to the other CS), and they obtained significantly different CPM effects based on their affinity. Accordingly, when they rated a high affinity for one CS, they obtained a significantly higher and more efficient CPM effect (CPM effect > 0.30), but when they showed a low/indifferent affinity for the other CS, applied obtained a less efficient CPM effect (CPM effect < 0.09). The remaining 55.56% shared similar affinities for both applied CS, so subjects with high affinity for both the cold water CS and the ischemia CS showed efficient CPM effects in both protocols without any difference between them. Similarly, subjects with low affinity for both stimuli also showed a low CPM effect without any significant difference between protocols. The unpleasantness variable within-subjects *t*-test revealed no significant variations in any combinations. The results of these analyses can be found in Table 3.

Exploring potential correlations between the additional variables and the CPM effect across both the ischemia and cold water protocols, we found a weak correlation between the extraversion variable and the CPM effect in the ischemia protocol and a strong correlation between CS duration and the CPM effect in the cold water protocol (Table 4). Furthermore, we analyzed the correlations between affinity and unpleasantness variables and CS duration. The analysis identified strong correlations between CS duration and both affinity (r = 0.56) and unpleasantness (r = −0.64) variables in the cold water protocol and moderate correlations with affinity (r = 0.36) and unpleasantness (r = −0.37) variables in the ischemia protocol.

Finally, the multiple linear regression analysis showed that the affinity variable was the main factor explaining the dependent variable CPM effect variance (Table 5). In the ‘Ischemia Protocol’, the affinity variable accounted for 27% of the explained variance in the CPM effect, while in the ‘Cold Water Protocol’, it accounted for 39% of the explained variance. The beta coefficients were positive (0.05 and 0.06), indicating that a higher affinity for the stimulus was associated with a stronger CPM effect. The unpleasantness variable and the additional variables assessed did not contribute to any explanatory models.

## 4. Discussion

The main objective of this study was to determine how variables such as affinity and unpleasantness impact the CPM effect using two different protocols (cold water and ischemia stimuli) in the same individuals. Additional variables were also measured to assess their potential influence on the CPM effect. The key findings of the study are as follows: (1) A higher level of affinity for the stimuli used resulted in a stronger CPM effect, and a lower level of unpleasantness for the stimuli led to a greater CPM effect; (2) Significant differences were observed in the extreme categories (high and low) of both affinity and unpleasantness variables, whereas the ‘indifferent’ group did not show a clear trend; (3) The within-subject analysis showed that affinity for CS clearly determines significant changes in the CPM effect; a strong affinity of a subject for a CS significantly influences their CPM effect; (4) No correlations were found between the CPM effect and the additional variables, except for the extraversion variable with the CPM effect of the ischemia protocol, and CS duration variable with CPM effect in the cold water protocol; (5) Finally, only the affinity variable explained the CPM effect in both protocols in the multiple linear regression analysis.

Previous studies have examined the characteristics of CS; specifically, the relationship between CS intensity and CPM has been extensively studied [14,15,16]. However, there is limited research on other characteristics like unpleasantness and affinity. A recent study investigated the attribute of pain unpleasantness and the impact on the CPM effect, finding that lower CS unpleasantness was associated with an improved CPM effect, possibly due to the maintenance of a negative state of arousal [11]. Regarding the affinity variable, there is one previous study where participants who received the cold water CS and had a higher affinity for the cold exhibited an inhibitory effect, while those without a preference for the cold did not show a significant CPM effect [17]. These results agree with our findings, where individuals with a higher level of affinity and a lower level of unpleasantness for the stimuli used resulted in a stronger CPM effect in both protocols. However, it is essential to note the difference between affinity and unpleasantness. Both variables belong to the cognitive–emotional dimension but differ in timing and experiential context. ‘Affinity’ refers to a pre-existing liking or aversion to a stimulus established previously; it is an opinion or belief created by a previous experience. On the other hand, ‘unpleasantness’ is felt during or after exposure to the CS, encompassing the immediate sensory and emotional reaction. Affinity (a belief created by a previous experience) could influence the CPM effect by predisposing the individual to develop an expectation (positive or negative) and subsequently a placebo or nocebo response, determining the effect of CPM (inhibition/facilitation). Previous studies have shown that individual belief in the current experience critically influences response expectancy through learning mechanisms [31,32]. Moreover, there is a potential interaction between emotions and expectations in pain processing [33]. Placebo and nocebo effects often involve emotional responses, such as anticipatory anxiety (nocebo) or positive feelings of relief and reward (placebo), which may mediate pain modulation [34,35,36]. In this context, one study explored the influence of expectations on the CPM effect, finding that a greater expectation of analgesia when applying a CS is associated with a greater capacity for CPM inhibition [37]. It is thus conceivable that affinity might similarly influence the CPM effect. Specifically, participants with lower affinity toward the stimulus might predisposed or anticipate more harm or discomfort (unpleasantness) from the CS applied, leading to a nocebo response and a consequently diminished CPM effect. Moreover, it is important to consider that the variables of affinity and unpleasantness are part of the affective or emotional dimensions of the pain matrix. Thus, brain structures such as the medial prefrontal cortex, premotor cortex, cingulate cortex, thalamus, and amygdala [38,39,40], as well as neurotransmitters like serotonin, dopamine, and norepinephrine [41,42,43], may mediate the CPM effect. This mediation has been supported by other research investigating the roles of harm avoidance [44], anxiety, depression, and pain catastrophizing in modulating the CPM effect [10].

In fact, these last-mentioned variables (anxiety, depression, and pain catastrophizing) are commonly studied [10]; however, our study found no correlations between these variables and the CPM effect. This lack of association may be because most of our participants had low levels of these variables, indicating that they did not generally experience these processes. In contrast, patients with chronic pain often have high levels of anxiety, depression, and catastrophism [45,46,47,48,49] and also tend to have poor CPM [6]. We also did not find correlations with additional variables such as resilience, kinesiophobia, stress, or personality traits like neuroticism, psychoticism, and sincerity. The relationship between some of these variables and the CPM effect is controversial and not as well-studied. For example, a study on healthy triathletes found that fear of pain is associated with less efficient CPM [50], but this association was not found in non-triathlete participants, and another study was conducted in healthy individuals [37]. Similarly, while a study in healthy individuals linked higher stress levels with less efficient CPM [51], another study did not find this association [52]. Studies have also shown no associations between neuroticism and the CPM effect [53,54]. In a study mentioned previously, men with inefficient CPM and higher resilience showed a higher CPM efficiency [11].

A notable weak correlation identified in our study was between the extraversion variable and the CPM effect in the ischemia protocol, where individuals with higher levels of extraversion exhibited a reduced CPM effect. These results may align with findings from imaging studies in healthy subjects, which have shown that extraversion is linked to increased activity in brain regions related to the nociceptive system [55,56]. The fact that this relationship was only found with the CPM effect ischemia protocol variable could be explained by evidence suggesting that the activation of pain inhibition processes differs depending on the modality of the applied stimulus. Specifically, pressure-based CPM effects were correlated with anxiety, heat-based CPM was correlated with depression, and electrical-based CPM was correlated with pain catastrophizing levels in healthy individuals [10].

In examining whether the CS duration variable correlates with the CPM effect in both protocols, a strong correlation was found in the cold water protocol but not in the ischemia protocol. Further analysis explored correlations between CS duration and the affinity and unpleasantness variables in both protocols. This analysis revealed strong correlations between CS duration and both affinity (r = 0.56) and unpleasantness (r = −0.64) in the cold water protocol and moderate correlations in the ischemia protocol. These results indicate that individuals with greater affinity for the stimulus and who rated the applied CS as less unpleasant sustained the CS application for longer durations. Thus, the duration of CS may also be influenced by the studied variables of stimulus affinity and unpleasantness, so the duration of CS alone does not determine the CPM effect. Moreover, while scientific evidence on how CS duration may influence the CPM effect is mixed and requires further investigation [5], some studies show that extending CS duration does not necessarily increase CPM magnitude [57].

Finally, multiple linear regression analysis found that only the affinity variable explained the dependent variable CPM effect, with 27% in the ‘Ischemia Protocol’ and 39% in the ‘Cold Water Protocol’. The Beta coefficients in both cases are positive, meaning a higher affinity for the stimulus leads to a higher CPM effect. The variable unpleasantness and the additional variables measured did not contribute to any explanatory model. The percentage varies depending on the applied CS, even though the same population was used. One possible explanation is that not all variables have the same influence on the CPM effect depending on their CS (as previously seen, studies suggest that the activation of pain inhibition processes differs depending on the modality of the applied stimulus) [10]. Conversely, a previous study found that the affinity variable explained 45% of the CPM effect for cold water [17]. The difference in percentages may be due to individual differences or methodological variations. These results reinforce the hypothesis that the affinity variable may determine the CPM effect more than other variables studied in healthy populations.

The findings suggest that cognitive–emotional variables must be considered when applying and interpreting CPM in clinical and research settings, particularly pre-existing inclinations or aversions toward specific stimuli (affinity). This could lead to more personalized approaches in pain management, where the cognitive–emotional profiles of individuals are considered, potentially improving the effectiveness and patient adherence to pain management interventions.

The study’s limitations include a relatively small sample size, which may limit the generalizability of the findings. Additionally, using different stimuli in CPM studies may yield different results, indicating the variability and specificity of CPM effects to different stimuli. Furthermore, the study only included healthy individuals, which may limit the applicability of the findings to clinical populations, e.g., those experiencing persistent pain. Further investigations with varied stimuli in diverse populations, including those experiencing persistent pain, are warranted to validate and expand upon these findings, enhancing their applicability and translatability to broader clinical contexts.

## 5. Conclusions

In conclusion, the affinity variable was found to influence the CPM effect significantly, indicating its significant role in our perception and response to pain. The findings emphasize the importance of considering individual psychological and emotional profiles when developing strategies for managing pain. They could have an influence on the development of future guidelines and affect the implementation of CPM in clinical settings.

## Figures and Tables

**Table 1:** Sociodemographic characteristics, psychological variables, and CPM effect outcomes.

Variables	Mean and SD or n (%) Total Cohort (n = 54)
Sex	
Male	19 (35.20)
Female	35 (68.80)
Age (years)	39.15 ± 13.81
BMI (kg/m^2^)	24.19 ± 3.89
HADS Total (0–42)	10.52 ± 5.93
HADS Depression (0–21)	3.31 ± 2.52
HADS Anxiety (0–21)	7.20 ± 3.96
PCS (0–52)	14.11 ± 10.79
BRS (6–30)	20.28 ± 4.37
PSS (0–56)	21.09 ± 8.31
TSK (11–44)	20.20 ± 5.59
Personality	
Extraversion (0–6)	4.31 ± 1.97
Neuroticism (0–6)	2.04 ± 1.80
Psychoticism (0–6)	2.35 ± 0.89
Sincerity (0–6)	2.91 ± 1.51
CPM Effect	
Cold Water Protocol	0.16 ± 0.31
Ischemia Protocol	0.17 ± 0.30
Duration CS (seconds)	
Cold Water	68.65 ± 39.34
Ischemia	121.26 ± 41.30

SD: Standard Deviation; %: Percentages; BMI: Body Mass Index; HADS: Hospital Anxiety and Depression Scale; PCS: Pain Catastrophizing; BRS: Brief Resilience Scale; PSS: Perceived Stress Scale; TSK: Tampa Scale Kinesiophobia; CPM: Conditioned Pain Modulation; CS: Conditioning Stimulus.

**Table 2:** Relationship between CPM effect and categorized affinity and unpleasantness variables in both protocols.

Stimulus	Variable	CPM Effect (Mean ± SD)	*p*-Value	Bonferroni Multiple Comparison Tests	*p*-Value
Cold Water (n = 54)	Affinity		0.00		
	Low (n = 16)	−0.11 ± 0.34		Low Affinity × Indifferent	0.00
	Indifferent (n = 16)	0.21 ± 0.18		Low Affinity × High Affinity	0.00
	High (n = 22)	0.32 ± 0.23		Indifferent × High Affinity	0.56
	Unpleasantness		0.00		
	Low (n = 11)	0.32 ± 0.18		Low Unpleasantness × Indifferent	1.00
	Indifferent (n = 20)	0.27 ± 0.31		Low Unpleasantness × High Unpleasantness	0.01
	High (n = 23)	0.00 ± 0.30		Indifferent × High Unpleasantness	0.01
Ischemia (n = 54)	Affinity		0.00		
	Low (n = 10)	0.01 ± 0.12		Low Affinity × Indifferent	0.84
	Indifferent (n = 32)	0.11 ± 0.18		Low Affinity × High Affinity	0.00
	High (n = 12)	0.50 ± 0.42		Indifferent × High Affinity	0.00
	Unpleasantness		0.00		
	Low (n = 15)	0.44 ± 0.40		Low Unpleasantness × Indifferent	0.00
	Indifferent (n = 29)	0.10 ± 0.17		Low Unpleasantness × High Unpleasantness	0.00
	High (n = 10)	0.01 ± 0.15		Indifferent × High Unpleasantness	1.00

CPM: Conditioned Pain Modulation.

**Table 3:** Intra-subject CPM effects according to affinity and unpleasantness.

Variables	Combinations	CPM Effect Cold Water Protocol	CPM Effect Ischemia Protocol	Mean Difference 95% CI	*p* Value
Affinity	High Affinity Cold Water and Indifferent/Low Affinity Ischemia (n = 17)	0.30 ± 0.26	0.09 ± 0.23	0.21 ± 0.30 (0.06–0.37)	0.01
	Indifferent/Low Affinity Cold Water and High Affinity Ischemia (n = 7)	−0.03 ± 0.22	0.59 ± 0.49	0.63 ± 0.61 (0.06–1.18)	0.04
	High Affinity Cold Water and High Affinity Ischemia (n = 5)	0.39 ± 0.14	0.38 ± 0.29	0.01 ± 0.24 (−0.28–0.31)	0.90
	Indifferent/Low Affinity Cold Water and Indifferent/Low Affinity Ischemia (n = 25)	0.07 ± 0.33	0.07 ± 0.13	−0.001 ± 0.61 (−0.13–0.13)	0.98
Unpleasantness	High Unpleasantness Cold Water and Indifferent/Low Unpleasantness Ischemia (n = 17)	−0.02 ± 0.34	0.07 ± 0.12	−0.10 ± 0.31 (−0.26 to 0.06)	0.21
	Indifferent/Low Unpleasantness Cold Water and High Unpleasantness Ischemia (n = 4)	0.24 ± 0.13	0.06 ± 0.18	0.18 ± 0.25 (−0.21 to 0.57)	0.24
	High Unpleasantness Cold Water and High Unpleasantness Ischemia (n = 6)	0.07 ± 0.10	−0.02 ± 0.13	0.09 ± 0.13 (−0.05 to 0.22)	0.15
	Indifferent/Low Unpleasantness Cold Water and Indifferent/Low Unpleasantness Ischemia (n = 27)	0.29 ± 0.28	0.30 ± 0.37	−0.1 ± 0.54 (−0.23 to 0.20)	0.91

CPM: Conditioned Pain Modulation; CI: Confidence Interval.

**Table 4:** Correlations between the additional variables and the CPM effect in ischemia and cold water protocols.

Variables (Score Range)	CPM Effect Cold Water (r Pearson)	CPM Effect Ischemia (r Pearson)
HADS Total (0–42)	−0.10	0.11
HADS Depression (0–21)	−0.06	0.13
HADS Anxiety (0–21)	−0.11	0.08
PCS (0–52)	0.00	−0.14
BRS (6–30)	0.04	0.05
PSS (0–56)	0.11	0.18
TSK (11–44)	−0.20	0.08
Extraversion (0–6)	−0.04	−0.27 *
Neuroticism (0–6)	−0.24	0.10
Psychoticism (0–6)	−0.06	0.14
Sincerity (0–6)	0.08	−0.06
CS Duration (seconds)	0.51 **	0.05

* *p* < 0.05; ** *p* < 0.01; CPM: Conditioned Pain Modulation; HADS; Hospital Anxiety and Depression Scale; PCS: Pain Catastrophizing Scale; BRS: Brief Resilience Scale; PSS: Perceived Stress Scale; TSK: Tampa Scale Kinesiophobia.

**Table 5:** Multiple linear regression analysis for CPM effect in the “Cold Water Protocol” and the “Ischemia Protocol”.

	Variables	B	Beta	95% CI for B	F	*p*-Value	R^2^	Adjusted R^2^
Cold Water Protocol	Affinity	0.046	0.421	(0.014 to 0.078 ) *			0.424	0.389
	Unpleasantness	−0.006	−0.050	(−0.047 to 0.034)	12.248	<0.001		
	CS Duration	0.114	0.269	(−0.010 to 0.239)				
Ischemia Protocol	Affinity	0.062	0.395	(0.014 to 0.110) *			0.309	0.267
	Unpleasantness	−0.018	−0.132	(−0.061 to 0.024)	7.446	<0.001		
	Extraversion	−0.037	−0.241	(−0.074 to 0.001)				

CS: Conditioning Stimulus; B: Unstandardized Coefficient; Beta: Standardized Coefficient; CI: Confidence Interval; * *p*-value < 0.01; R^2^: R Square.

## Data Availability

Data used to support the findings of this study are available from the corresponding author upon request.

## References

[1] Yarnitsky D., Arendt-Nielsen L., Bouhassira D., Edwards R.R., Fillingim R.B., Granot M., Hansson P., Lautenbacher S., Marchand S., Wilder-Smith O. (2010). Recommendations on Terminology and Practice of Psychophysical DNIC Testing. Eur. J. Pain.

[2] Le Bars D., Dickenson A.H., Besson J.M. (1979). Diffuse Noxious Inhibitory Controls (DNIC). I. Effects on Dorsal Horn Convergent Neurones in the Rat. Pain.

[3] Bannister K., Dickenson A.H. (2017). The Plasticity of Descending Controls in Pain: Translational Probing. J. Physiol..

[4] Villanueva L. (2009). Diffuse Noxious Inhibitory Control (DNIC) as a Tool for Exploring Dysfunction of Endogenous Pain Modulatory Systems. Pain.

[5] Damien J., Colloca L., Bellei-Rodriguez C.É., Marchand S. (2018). Pain Modulation: From Conditioned Pain Modulation to Placebo and Nocebo Effects in Experimental and Clinical Pain. International Review of Neurobiology.

[6] Lewis G.N., Rice D.A., McNair P.J. (2012). Conditioned Pain Modulation in Populations with Chronic Pain: A Systematic Review and Meta-Analysis. J. Pain.

[7] Hermans L., Van Oosterwijck J., Goubert D., Goudman L., Crombez G., Calders P., Meeus M. (2016). Inventory of Personal Factors Influencing Conditioned Pain Modulation in Healthy People: A Systematic Literature Review. Pain Pract..

[8] Skovbjerg S., Jørgensen T., Arendt-Nielsen L., Ebstrup J.F., Carstensen T., Graven-Nielsen T. (2017). Conditioned Pain Modulation and Pressure Pain Sensitivity in the Adult Danish General Population: The DanFunD Study. J. Pain.

[9] Klyne D.M., Moseley G.L., Sterling M., Barbe M.F., Hodges P.W. (2018). Individual Variation in Pain Sensitivity and Conditioned Pain Modulation in Acute Low Back Pain: Effect of Stimulus Type, Sleep, and Psychological and Lifestyle Factors. J. Pain.

[10] Nahman-Averbuch H., Nir R.-R., Sprecher E., Yarnitsky D. (2016). Psychological Factors and Conditioned Pain Modulation. Clin. J Pain.

[11] Firouzian S., Osborne N.R., Cheng J.C., Kim J.A., Bosma R.L., Hemington K.S., Rogachov A., Davis K.D. (2020). Individual Variability and Sex Differences in Conditioned Pain Modulation and the Impact of Resilience, and Conditioning Stimulus Pain Unpleasantness and Salience. Pain.

[12] Geers A.L., Wellman J.A., Fowler S.L., Helfer S.G., France C.R. (2010). Dispositional Optimism Predicts Placebo Analgesia. J. Pain.

[13] France C.R., Burns J.W., Gupta R.K., Buvanendran A., Chont M., Schuster E., Orlowska D., Bruehl S. (2016). Expectancy Effects on Conditioned Pain Modulation Are Not Influenced by Naloxone or Morphine. Ann. Behav. Med..

[14] Willer J.C., De Broucker T., Le Bars D. (1989). Encoding of Nociceptive Thermal Stimuli by Diffuse Noxious Inhibitory Controls in Humans. J. Neurophysiol..

[15] Nir R.R., Granovsky Y., Yarnitsky D., Sprecher E., Granot M. (2011). A Psychophysical Study of Endogenous Analgesia: The Role of the Conditioning Pain in the Induction and Magnitude of Conditioned Pain Modulation. Eur. J. Pain.

[16] Nir R.R., Yarnitsky D., Honigman L., Granot M. (2012). Cognitive Manipulation Targeted at Decreasing the Conditioning Pain Perception Reduces the Efficacy of Conditioned Pain Modulation. Pain.

[17] Ibancos-Losada M.D.R., Osuna-Pérez M.C., Castellote-Caballero M.Y., Díaz-Fernández Á. (2020). Conditioned Pain Modulation Effectiveness: An Experimental Study Comparing Test Paradigms and Analyzing Potential Predictors in a Healthy Population. Brain Sci..

[18] Matre D. (2013). Conditioned Pain Modulation (CPM) Is Not One Single Phenomenon—Large Intra-Individual Differences Depend on Test Stimulus (TS) and Several Other Independent Factors. Scand. J. Pain.

[19] Kennedy D.L., Kemp H.I., Ridout D., Yarnitsky D., Rice A.S.C. (2016). Reliability of Conditioned Pain Modulation. Pain.

[20] Corrêa J.B., Costa L.O.P., de Oliveira N.T.B., Sluka K.A., Liebano R.E. (2015). Central Sensitization and Changes in Conditioned Pain Modulation in People with Chronic Nonspecific Low Back Pain: A Case–Control Study. Exp. Brain Res..

[21] Fernandez-Carnero J., Sierra-Silvestre E., Beltran-Alacreu H., Gil-Martınez A., La Touche R. (2019). Neural Tension Technique Improves Immediate Conditioned Pain Modulation in Patients with Chronic Neck Pain: A Randomized Clinical Trial. Pain Med..

[22] Yarnitsky D., Bouhassira D., Drewes A.M., Fillingim R.B., Granot M., Hansson P., Landau R., Marchand S., Matre D., Nilsen K.B. (2015). Recommendations on Practice of Conditioned Pain Modulation (CPM) Testing. Eur. J. Pain.

[23] Boonstra A.M., Preuper H.R.S., Balk G.A., Stewart R.E. (2014). Cut-off Points for Mild, Moderate, and Severe Pain on the Visual Analogue Scale for Pain in Patients with Chronic Musculoskeletal Pain. Pain.

[24] Herrero M.J., Blanch J., Peri J.M., De Pablo J., Pintor L., Bulbena A. (2003). A Validation Study of the Hospital Anxiety and Depression Scale (HADS) in a Spanish Population. Gen. Hosp. Psychiatry.

[25] Remor E. (2006). Psychometric Properties of a European Spanish Version of the Perceived Stress Scale (PSS). Span. J. Psychol..

[26] García Campayo J., Rodero B., Alda M., Sobradiel N., Montero J., Moreno S. (2008). Validation of the Spanish Version of the Pain Catastrophizing Scale in Fibromyalgia. Med. Clin..

[27] Gómez-Pérez L., López-Martínez A.E., Ruiz-Párraga G.T. (2011). Psychometric Properties of the Spanish Version of the Tampa Scale for Kinesiophobia (TSK). J. Pain.

[28] Rodríguez-Rey R., Alonso-Tapia J., Hernansaiz-Garrido H. (2016). Reliability and Validity of the Brief Resilience Scale (BRS) Spanish Version. Psychol. Assess.

[29] Sandín B., Valiente R.M., Olmedo Montes M., Chorot P., Santed Germán M.A. (2002). Versión Española Del Cuestionario EPQR-ABREVIADO (EPQR-A) (II): Replicación Factorial, Fiabilidad y Validez. Rev. De Psicopatología Psicol. Clínica.

[30] Cohen J. (2013). Statistical Power Analysis for the Behavioral Sciences.

[31] Enck P., Bingel U., Schedlowski M., Rief W. (2013). The Placebo Response in Medicine: Minimize, Maximize or Personalize?. Nat. Rev. Drug Discov..

[32] Kirsch I., Weixel L.J. (1988). Double-Blind Versus Deceptive Administration of a Placebo. Behav. Neurosci..

[33] Reicherts P., Pauli P., Mösler C., Wieser M.J. (2019). Placebo Manipulations Reverse Pain Potentiation by Unpleasant Affective Stimuli. Front. Psychiatry.

[34] Benedetti F., Amanzio M., Vighetti S., Asteggiano G. (2006). The Biochemical and Neuroendocrine Bases of the Hyperalgesic Nocebo Effect. J. Neurosci..

[35] Flaten M.A., Aslaksen P.M., Lyby P.S., Bjørkedal E. (2011). The Relation of Emotions to Placebo Responses. Philos. Trans. R. Soc. B Biol. Sci..

[36] Scott D.J., Stohler C.S., Egnatuk C.M., Wang H., Koeppe R.A., Zubieta J.K. (2008). Placebo and Nocebo Effects Are Defined by Opposite Opioid and Dopaminergic Responses. Arch. Gen. Psychiatry.

[37] Bjørkedal E., Flaten M.A. (2012). Expectations of Increased and Decreased Pain Explain the Effect of Conditioned Pain Modulation in Females. J. Pain Res..

[38] Ziv M., Tomer R., Defrin R., Hendler T. (2010). Individual Sensitivity to Pain Expectancy Is Related to Differential Activation of the Hippocampus and Amygdala. Hum. Brain Mapp..

[39] Sprenger C., Bingel U., Büchel C. (2011). Treating Pain with Pain: Supraspinal Mechanisms of Endogenous Analgesia Elicited by Heterotopic Noxious Conditioning Stimulation. Pain.

[40] Piché M., Arsenault M., Rainville P. (2009). Cerebral and Cerebrospinal Processes Underlying Counterirritation Analgesia. J. Neurosci..

[41] Treister R., Pud D., Eisenberg E. (2013). The Dopamine Agonist Apomorphine Enhances Conditioned Pain Modulation in Healthy Humans. Neurosci. Lett..

[42] Baba Y., Kohase H., Oono Y., Fujii-Abe K., Arendt-Nielsen L. (2012). Effects of Dexmedetomidine on Conditioned Pain Modulation in Humans. Eur. J. Pain.

[43] Lindstedt F., Berrebi J., Greayer E., Lonsdorf T.B., Schalling M., Ingvar M., Kosek E. (2011). Conditioned Pain Modulation Is Associated with Common Polymorphisms in the Serotonin Transporter Gene. PLoS ONE.

[44] Nahman-Averbuch H., Yarnitsky D., Sprecher E., Granovsky Y., Granot M. (2016). Relationship between Personality Traits and Endogenous Analgesia: The Role of Harm Avoidance. Pain Pract..

[45] Lackner J.M., Quigley B.M., Blanchard E.B. (2004). Depression and Abdominal Pain in IBS Patients: The Mediating Role of Catastrophizing. Psychosom. Med..

[46] Smith B.W., Tooley E.M., Montague E.Q., Robinson A.E., Cosper C.J., Mullins P.G. (2008). Habituation and Sensitization to Heat and Cold Pain in Women with Fibromyalgia and Healthy Controls. Pain.

[47] Prasko J., Jelenova D., Mihal V. (2010). Psychological Aspects and Psychotherapy of Inflammatory Bowel Diseases and Irritable Bowel Syndrome in Children. Biomed. Pap..

[48] Burgmer M., Petzke F., Giesecke T., Gaubitz M., Heuft G., Pfleiderer B. (2011). Cerebral Activation and Catastrophizing during Pain Anticipation in Patients with Fibromyalgia. Psychosom. Med..

[49] Rist P.M., Schürks M., Buring J.E., Kurth T. (2013). Migraine, Headache, and the Risk of Depression: Prospective Cohort Study. Cephalalgia.

[50] Geva N., Defrin R. (2013). Enhanced Pain Modulation among Triathletes: A Possible Explanation for Their Exceptional Capabilities. Pain.

[51] Wilder-Smith C.H., Song G., Yeoh K.G., Ho K.Y. (2009). Activating Endogenous Visceral Pain Modulation: A Comparison of Heterotopic Stimulation Methods in Healthy Controls. Eur. J. Pain.

[52] Heymen S., Maixner W., Whitehead W.E., Klatzkin R.R., Mechlin B., Light K.C. (2010). Central Processing of Noxious Somatic Stimuli in Patients with Irritable Bowel Syndrome Compared with Healthy Controls. Clin. J. Pain.

[53] van Laarhoven A.I.M., Kraaimaat F.W., Wilder-Smith O.H., van de Kerkhof P.C.M., Evers A.W.M. (2010). Heterotopic Pruritic Conditioning and Itch—Analogous to DNIC in Pain?. Pain.

[54] Hinkle C.E., Quiton R.L. (2019). Higher Dispositional Optimism Predicts Lower Pain Reduction During Conditioned Pain Modulation. J. Pain.

[55] Ruffle J.K., Farmer A.D., Kano M., Giampietro V., Aziz Q., Coen S.J. (2015). The Influence of Extraversion on Brain Activity at Baseline and during the Experience and Expectation of Visceral Pain. Pers. Individ. Dif..

[56] Lei X., Yang T., Wu T. (2015). Functional Neuroimaging of Extraversion-Introversion. Neurosci. Bull..

[57] Smith A., Pedler A. (2018). Conditioned Pain Modulation Is Affected by Occlusion Cuff Conditioning Stimulus Intensity, but Not Duration. Eur. J. Pain.

